# The gut-brain axis in Alzheimer’s disease: traditional Chinese medicine constituents as modulators of gut-brain homeostasis

**DOI:** 10.3389/fmicb.2026.1852198

**Published:** 2026-08-27

**Authors:** Yichao Liang, Qian Kong, Xiao Du, Yunting Sun, Shuling Wang

**Affiliations:** 1 School of Pharmacy, Zhejiang Provincial Key Laboratory of Anti-Cancer Chinese Medicines and Natural Medicines, Hangzhou Normal University, Hangzhou, Zhejiang, China; 2 Hangzhou Traditional Chinese Medicine Hospital Affiliated to Zhejiang Chinese Medical University, Hangzhou, Zhejiang, China

**Keywords:** Alzheimer’s disease, gut microbiota-gut-brain axis, microglia, neuroinflammation, short-chain fatty acids, traditional Chinese medicine

## Abstract

Alzheimer’s disease (AD) currently lacks curative treatments, and the gut–brain axis plays an important role in regulating neuroinflammation during AD progression. Multiple bioactive components from traditional Chinese medicine (TCM), including polysaccharides, alkaloids, flavonoids and saponins, protect neurons through regulating gut microbiota composition, maintaining intestinal barrier function and modulating short-chain fatty acid (SCFA) metabolism. Different from previous reviews that mainly elaborate correlational relationships, the present paper systematically sorts out a multi-level action network of TCM components along the gut-brain axis. We address an interesting discrepancy in current findings on SCFAs: acetate can inhibit inflammation in microglia under mechanical stretch, yet fails to produce the same effect under LPS exposure. Nevertheless, increased SCFA levels *in vivo* are able to alleviate microglial dysfunction linked to aging. Such findings indicate that SCFA activities are influenced by stimulus types, compound categories and applied doses, reminding researchers to treat existing conclusions with caution. We also outline six widely existing methodological issues in relevant researches, and propose feasible solutions, including the documentation of absolute SCFA concentrations, experimental verification of causal relationships, and the use of AD pathological stimuli like Aβ and tau. This review may facilitate the shift from correlational analysis to mechanistic exploration, and provide useful references for the development of AD therapies targeting the gut–brain axis with TCM components.

## 1 Background and current status of AD

AD is one of the most common neurodegenerative disorders, primarily characterized by progressive memory loss, cognitive impairment, and a range of distressing symptoms (Long et al., 2025; Shen et al., 2025; Cui et al., 2021). The etiology and pathogenesis of AD are highly complex and exhibit considerable heterogeneity. Despite extensive research over the years, the underlying mechanisms of the disease remain unclear (Xie et al., 2025; Shou et al., 2020; Shou et al., 2020; Zhu et al., 2020). The typical pathological hallmarks of AD include the abnormal aggregation of hyperphosphorylated tau protein within neurons, leading to the formation of neurofibrillary tangles, as well as the deposition of extracellular *β*-amyloid (Aβ) plaques. Aβ plaques and neurofibrillary tangles may further exacerbate tau hyperphosphorylation and aggregation by inducing synaptic dysfunction. The abnormal accumulation of Aβ in the brain promotes the generation of large amounts of reactive oxygen species (ROS), which in turn cause oxidative damage to biomolecules such as DNA, lipids, and proteins (Ji et al., 2025; Li J. Z. et al., 2025; Ding et al., 2024; Gonçalves et al., 2024). Moreover, abnormal extracellular and intracellular deposits in the brain can trigger inflammatory responses, resulting in a series of pathological changes, including disruption of the blood–brain barrier (BBB), metabolic dysfunction, and brain atrophy (Preethy et al., 2024). The incidence and prevalence of AD increase sharply with age, with approximately 80% of patients being over 75 years old (Wanionok et al., 2024). This age-related trend may be associated with the decline in androgen secretion that occurs during aging. Lower androgen levels can induce apoptosis in microglia and impair their proliferative capacity, thereby accelerating the pathological progression of AD (Cao et al., 2024). According to data from the World Health Organization (WHO), as of 2023, 55 million people worldwide were living with dementia. As the population continues to age, this number is projected to surge to 139 million by 2050. AD is the leading cause of dementia, accounting for more than two-thirds of all cases (Hou et al., 2024). Given the complexity and diversity of AD pathophysiology, the number of clinically available treatments is extremely limited. To date, only five drugs—tacrine, donepezil, rivastigmine, galantamine, and memantine—have been approved by the U.S. Food and Drug Administration (FDA) for the treatment of AD. Although these drugs can ameliorate symptoms to some extent, they cannot fundamentally halt disease progression (Li J. et al., 2024; Binda et al., 2020). Therefore, the development of new therapeutic agents for AD has become particularly urgent.

## 2 Basic structure and communication pathways of the microbiota-gut-brain axis

The microbiota-gut-brain axis is a complex, bidirectional signaling network that closely links the central nervous system, the gut, and its resident microbial communities. Gut microbes continuously communicate with the brain through multiple pathways, including neural, immune, endocrine, and metabolic mechanisms (Cryan et al., 2019; Doifode et al., 2021; Giridharan et al., 2022; He Y. et al., 2024; Zhong et al., 2021; Faulin and Estadella, 2023; Liu et al., 2020; Yao et al., 2020; Zhao et al., 2022).

In terms of neural pathways, the vagus nerve and the enteric nervous system form a direct “neural highway” (Cryan et al., 2019; Giridharan et al., 2022). Regarding immune and inflammatory pathways, the gut microbiota regulates local and systemic immune responses; gut dysbiosis can impair the intestinal barrier, allowing bacterial products such as LPS to enter the bloodstream and trigger systemic inflammation. In turn, systemic inflammation can cross the BBB and activate microglia (Doifode et al., 2021; Qu et al., 2024; Megur et al., 2020; Lin et al., 2019). Through endocrine pathways, the gut microbiota influences the hypothalamic–pituitary–adrenal (HPA) axis, thereby modulating the body’s stress response (Giridharan et al., 2022). Among these, metabolic pathways are particularly critical: gut microbiota ferment dietary fiber to produce SCFAs, such as butyrate, which participate in tryptophan metabolism to generate serotonin or kynurenine pathway metabolites. These microbial metabolites can enter the circulatory system and directly act on the BBB and neurons (Cryan et al., 2019; Doifode et al., 2021; Zhong et al., 2021; Doifode et al., 2021; Escobar et al., 2022).

## 3 The link between dysbiosis and AD pathology

During the course of AD, the homeostasis of this axis is disrupted, leading to a state known as “gut-brain axis dysregulation,” which drives disease progression at multiple levels (Qu et al., 2024). Changes in microbial community structure: dysbiosis of the gut microbiota is commonly observed in patients with AD. Compared to healthy individuals, the abundance of *Clostridia*_UCG-014 is elevated, whereas the abundance of beneficial bacteria such as *Moryella* and *Blautia* is reduced (Giridharan et al., 2022; Marizzoni et al., 2026; Marizzoni et al., 2023). These changes are closely associated with disease severity and pathological markers (Marizzoni et al., 2026; Marizzoni et al., 2023).

Leaky gut and systemic inflammation: Gut microbiota imbalance leads to increased intestinal barrier permeability (i.e., “leaky gut”), allowing pro-inflammatory substances such as LPS to enter the bloodstream and trigger systemic low-grade inflammation. These inflammatory signals are transmitted to the brain, activating microglia and inducing excessive neuroinflammatory responses (Qu et al., 2024; Megur et al., 2020; Cui et al., 2024). Patients with AD exhibit elevated blood levels of LPS and pro-inflammatory cytokines (IL-1β, IL-6, TNF-*α*), which are associated with alterations in the gut microbiota (Marizzoni et al., 2023).

Amyloid and tau pathology: Gut microbiota and their metabolites modulate host immunity, influencing the clearance and deposition of Aβ in the brain (Doifode et al., 2021). Certain bacteria can also produce amyloid proteins autonomously, which may contribute to plaque formation in the brain (Megur et al., 2020). Preclinical studies have shown that modulating the gut microbiota via fecal microbiota transplantation can correct tau hyperphosphorylation (Ren et al., 2025).

Metabolic disorders: dysbiosis disrupts tryptophan metabolism, shifting it from serotonin production toward the generation of neurotoxic kynurenine pathway metabolites (e.g., quinolinic acid) (Cui et al., 2024; Wang J. et al., 2026). Similarly, disruption of the arginine biosynthetic pathway leads to a reduction in neuroprotective metabolites (such as L-citrulline and L-arginine), further exacerbating neuroinflammation (Wang J. et al., 2026). Finally, ecological imbalance not only damages the intestinal barrier but may also compromise the integrity of the blood–brain barrier through mechanisms involving inflammatory factors, making it easier for harmful peripheral substances to enter the brain and creating a vicious cycle (Zhong et al., 2021).

## 4 The advantages of active compounds in TCM as “chemical tools” for modulating the gut microbiome

Active compounds in TCM, such as flavonoids, triterpenes, and alkaloids, exert multi-target synergistic effects. They can simultaneously modulate gut microbiota imbalance, suppress neuroinflammation, and reduce Aβ deposition and tau hyperphosphorylation, thereby comprehensively intervening in the pathological progression of AD (Ma et al., 2023; He et al., 2023; Shen et al., 2024; Zhang and Park, 2025). For example, berberine promotes the proliferation of *Bifidobacterium* via the SCFAs pathway, thereby improving cognitive function (Xie et al., 2023). Ganoderma triterpenes modulate the balance of gut microbiota and metabolites (e.g., aromatic amino acid metabolism) and inhibit oxidative stress (Shen et al., 2024). In addition, TCM compounds (e.g., curcumin, ginkgolides, and ginsenosides) exhibit structural diversity and high biocompatibility; they penetrate the BBB more easily than synthetic drugs and have fewer side effects (Pavlov et al., 2025; Zhan et al., 2023). For instance, curcumin alleviates neuroinflammation by modulating the NF-κB pathway while simultaneously modulating the gut microbiota (e.g., increasing *lactobacilli*) (Pavlov et al., 2025; Kim et al., 2024). *Ginkgo biloba* extract enhances synaptic plasticity via the BDNF/CREB pathway and modulates immune responses in the gut-brain axis (Pavlov et al., 2025).

More importantly, the active components of TCM can bidirectionally regulate the interaction between gut microbiota metabolites (such as SCFAs and tryptophan metabolites) and the central nervous system. In the SCFAs pathway, TCM components (e.g., from *Coptis*) increase the production of SCFAs such as butyrate, which in turn inhibit excessive microglial activation and reduce pro-inflammatory factors including IL-6 and TNF-*α* (Xie et al., 2023; Gu et al., 2024). In the tryptophan–kynurenine pathway, TCM formulas (e.g., Danggui Shaoyao San) modulate this metabolic route to reduce the accumulation of neurotoxic metabolites (He et al., 2023; Gu et al., 2024). At the same time, the active ingredients in TCM exert neuroprotective effects by inhibiting oxidative stress and modulating the immune system. For example, Danggui Shaoyao San increases SOD and GSH-PX activity and reduces MDA levels (He et al., 2023; Zhang and Park, 2025). Phytochemicals such as resveratrol reduce systemic inflammation by modulating the Th1/Th2 balance (Kim et al., 2024; Gu et al., 2024; Fang et al., 2026).

## 5 Recent progress in TCM monomer-based regulation of the gut-brain axis for AD therapy

AD is a progressive neurodegenerative disorder characterized by complex pathological mechanisms closely associated with gut microbiota dysbiosis. In recent years, increasing research has focused on the central role of the microbiota–gut–brain axis in the onset and progression of AD. In this context, the use of active monomeric compounds from TCM to treat AD by modulating the gut microbiota has emerged as a frontier research direction (Pasupalak et al., 2024; Zhao J. et al., 2026; Zhou et al., 2025). Several studies have revealed the potential of specific TCM monomers in treating AD via the gut-brain axis. For example, Xanthoceraside, a triterpenoid saponin extracted from the husk of *Xanthoceras sorbifolium*, has been shown to directly or indirectly correct Aβ-induced metabolic disturbances in gut neurotransmitters, amino acids, bile acids, and SCFAs by regulating the structure of gut microbial communities. This occurs despite its poor water solubility and limited ability to cross the BBB, ultimately leading to significant improvements in learning and memory deficits in AD rats (Zhou et al., 2022). Another example is uridine, a component derived from Polygonatum (Huangjing), which can enter brain tissue and alleviate Aβ deposition by modulating the abundance and diversity of the gut microbiota. Notably, the raw product of Polygonatum primarily acts by regulating inflammatory pathways, whereas the processed product focuses more on balancing cellular autophagy and apoptosis (Xiaojuan et al., 2024). Modern clinical studies have begun to validate these findings. In a randomized controlled trial, Bushen Yinao Pill combined with conventional treatment significantly increased the abundance of beneficial bacteria in the gut of AD patients, reduced harmful bacteria, downregulated levels of inflammatory markers including Aβ, IL-6, and TNF-*α*, and markedly improved patients’ cognitive function (Wang W. et al., 2025). Furthermore, substantial evidence suggests that TCM may also exert beneficial effects on AD treatment through multiple direct or indirect pathways (Wu et al., 2024; Gao et al., 2023; Yan et al., 2024; Chen et al., 2024; Hu et al., 2023; Tao et al., 2024; Han et al., 2024; Jin et al., 2022; Song et al., 2022; Zhu et al., 2021). These results indicate that TCM therapies based on gut–brain axis regulation are clinically feasible.

## 6 Classification and mechanisms of action of active compounds in TCM

### 6.1 Polysaccharides

Eucommia bark polysaccharides (EUPs) are natural macromolecules with diverse biological activities, including antioxidant, anti-inflammatory, immunomodulatory, and neuroprotective effects (Yu et al., 2024; Sun Y. et al., 2024). Recent studies have shown that EUPs can indirectly influence central nervous system function by modulating the gut–microbiota–brain axis. Specifically, EUPs regulate the gut microbiota and inhibit neuroinflammation. Regarding the maintenance of gut microbiota balance, EUPs significantly increase the abundance of beneficial bacteria (e.g., *Lactobacillaceae* and *Firmicutes*) and reduce that of harmful bacteria (e.g., *Helicobacter* and *Clostridium*) (Peng et al., 2024; Zhang et al., 2025). This modulation reduces intestinal permeability, limiting the entry of pro-inflammatory factors such as LPS into the bloodstream and thereby suppressing systemic inflammatory responses.

In terms of promoting SCFA production, EUPs enhance the synthesis of short-chain fatty acids (e.g., butyrate) by stimulating the growth of butyrate-producing bacteria such as *Butyricicoccus* (Zhang et al., 2025). The generated SCFAs can cross the BBB to directly inhibit excessive microglial activation, thereby reducing AD-related neuroinflammation (Ye et al., 2023). At the same time, EUPs repair the intestinal mucosal barrier by upregulating the expression of tight junction proteins (occludin, claudin-1, and ZO-1), enhancing intestinal barrier function and preventing enteric toxins from entering the circulatory system (Li Y. et al., 2024).

It is worth noting that EUPs may also play a role in neuroimmunomodulation and the protection of synaptic plasticity. Specifically, in AD models, EUPs promote the conversion of microglia to an anti-inflammatory (M2) phenotype, inhibit the release of pro-inflammatory cytokines (e.g., IL-6 and IL-1*β*), and simultaneously increase the expression of neurotrophic factors (e.g., TGF-β and BMP-6) (Sun et al., 2021; Deng et al., 2019). By modulating gut microbiota-derived metabolites (e.g., tryptophan metabolites), EUPs may indirectly influence brain levels of 5-HT and BDNF, thereby improving synaptic plasticity and cognitive function (Zhang et al., 2025; Song et al., 2023).

A study using 5 × FAD mice as an experimental model evaluated the effects of EUPs on AD mice. The results showed that EUPs enhance the proliferation of butyrate-producing bacteria in the gut, leading to butyrate production, which regulates the activity and metabolism of glutamine synthetase. This regulation promotes the balance of brain redox status and facilitates autophagy (Zhao Y. et al., 2026). Although the above study proposes an integrated EUPs–butyrate–glutamine synthetase–autophagy–Aβ clearance cascade, causal evidence remains incomplete. As elaborated in Section 7, the assumption that short-chain fatty acids universally suppress neuroinflammation under AD-relevant conditions requires direct validation, and the contribution of butyrate to cognitive improvement independent of other microbial metabolites has not been dissected.

Pseudostellaria heterophylla polysaccharide (PH-PS) is a plant-derived polysaccharide. Studies have shown that PH-PS can improve cognitive function in animal models of AD, alleviate brain pathological changes, and modulate the gut microbiota, thereby exerting a neuroprotective effect (He C. et al., 2024). The core of this neuroprotective effect lies in its regulatory role in the gut–brain axis. While promoting the growth of beneficial bacteria such as Lactobacillus, Muribaculum, Monoglobus, and the Lactococcus siraeum group, PH-PS inhibits the growth of inflammation-associated genera such as UCG-009 and Blautia. Furthermore, PH-PS helps restore the integrity of the damaged intestinal barrier, which is a crucial foundation for maintaining normal gut–brain axis communication and preventing peripheral inflammatory factors from entering the brain. By modulating the gut microbiota, PH-PS reduces the secretion of peripheral inflammatory cytokines; this anti-inflammatory effect is further transmitted to the brain, promoting the conversion of pro-inflammatory M1-type microglia and A1-type astrocytes into protective M2-type and A2-type cells (He C. et al., 2024).

Asparagus cochinchinensis polysaccharides (asparagus polysaccharides) are natural polysaccharide compounds extracted from Asparagus cochinchinensis. Studies have shown that asparagus acid polysaccharides can increase the abundance of beneficial bacteria such as *Lactobacillus gasseri* and members of the *Lachnospiraceae* family while simultaneously inhibiting the proliferation of pathogenic bacteria. In addition, asparagus acid polysaccharides restore microbial metabolic functions, particularly the synthesis of bile acids and SCFAs (Chen et al., 2023). These effects play a positive role in the prevention and treatment of AD.

### 6.2 Alkaloids

Berberine (BBR) is a natural isoquinoline alkaloid and the most common alkaloid in TCM. BBR can modulate the gut–brain axis through multiple mechanisms to alleviate AD pathology. Specifically, BBR increases the abundance of SCFA-producing bacteria (e.g., *Butyricimonas* and *Ruminococcus*) and *Bifidobacterium*, and elevates SCFA levels (e.g., acetate and indole-3-lactic acid); the latter exerts neuroprotective effects by inhibiting histone deacetylases (HDACs) (Xie et al., 2023). SCFAs (e.g., butyrate) can also inhibit the NF-κB pathway via GPR41/43 receptors, thereby reducing microglial activation and alleviating Aβ-induced neurotoxicity (Sun C. et al., 2024). In addition, BBR reduces the abundance of LPS-producing bacteria such as those in the genus *Arcobacter*, decreases the release of pro-inflammatory cytokines (IL-1β, TNF-*α*), and thereby alleviates systemic and neuroinflammation. It also upregulates the expression of mucin-2 and modulates tight junction proteins, reducing intestinal permeability and preventing endotoxins from entering the bloodstream (Sun C. et al., 2024).

Multiple animal studies have confirmed the efficacy of BBR in improving cognitive function. In various AD mouse models, BBR treatment significantly improved spatial learning and memory abilities, as demonstrated by behavioral tests such as the Morris water maze, novel object recognition, and Y-maze (Sun C. et al., 2024; Zhao et al., 2023; Lv et al., 2025; Wei et al., 2023). Despite the promising efficacy of berberine in animal models, its translation into clinical AD therapy faces substantial challenges. Due to its pharmacokinetic properties, the oral bioavailability of berberine is extremely low, creating a notable contradiction with its actual therapeutic effects. Berberine relies on gut microbiota to convert it into dihydroberberine, a polar form that is readily absorbed into the bloodstream, to exert systemic effects. This dependence on the host gut microbiota implies that interindividual differences in microbial composition can greatly affect drug conversion efficiency, making the predictability and consistency of clinical outcomes difficult to guarantee. Moreover, most human AD patients have a long disease course with multiple comorbidities, which is markedly different from the rapidly induced pathological processes in animal models. Therefore, future clinical translation may require multidisciplinary collaboration.

Matrine is a natural alkaloid. Recent studies have shown that it can improve neuropsychiatric disorders by regulating the microbiota–gut–brain axis. In a chronic unpredictable mild stress (CUMS) mouse model, matrine restored gut microbiota diversity, reduced the proliferation of pathogenic bacteria, increased the abundance of beneficial bacteria, lowered serum diamine oxidase (DAO) and LPS levels, alleviated intestinal permeability, and inhibited the migration of inflammatory cytokines (e.g., TNF-*α* and IL-1β) to the central nervous system (Zhang et al., 2023). In addition, matrine may protect neurons by inhibiting the kynurenine (Kyn) pathway, thereby reducing the accumulation of neurotoxic metabolites such as quinolinic acid (Deng et al., 2021; Bao et al., 2022; Tiwari and Paramanik, 2025).

Palmatine is one of the primary active components in TCM herbs such as Coptis chinensis and belongs to the isoquinoline alkaloid class. Studies have shown that palmatine can significantly increase the abundance of SCFA-producing bacterial populations (e.g., *Ruminococcus* and related species) and *Bifidobacterium breve*, thereby elevating SCFA levels. Increased SCFAs (e.g., acetate and indole-3-lactic acid) can activate the AMPK/NF-κB pathway in the brain via the BBB or vagal nerve signaling, thereby suppressing neuroinflammation and reducing Aβ deposition (Xie et al., 2023).

One study (Zhong et al., 2025) used three transgenic mouse models—TgCRND8 (carrying double APP mutations), 3 × Tg-AD (carrying APP/PS1/tau triple mutations), and 5 × FAD (carrying APP/PS1 quintuple mutations)—to simulate different AD pathological processes and validate the therapeutic potential of rhynchophylline (RN). The results showed that in the 5 × FAD model, which bears the most severe AD burden, RN restored the Firmicutes/Bacteroidetes ratio to wild-type levels and increased the abundance of beneficial bacteria such as Bifidobacterium by approximately two-fold. These findings further support the role of gut microbiota remodeling in the gut–brain axis regulation of AD. Another study (Ni et al., 2024) used scopolamine-induced AD model mice to investigate how sinomenine maintains brain and gut homeostasis by modulating cationic antimicrobial peptides (CAPs). The results showed that the ability of CAPs to inhibit the TLR4/NF-κB pathway was impaired in model mice, whereas sinomenine restored this function, thereby protecting brain and gut homeostasis and alleviating cognitive impairment.

### 6.3 Flavonoids

Andrographolide is a common flavonoid compound found in TCM. Studies have shown that andrographolide treatment can effectively reverse cognitive impairment in APP/PS1 mice while also reducing pathological Aβ deposits and neuroinflammation in the mice’s brains (Zhang S. et al., 2022). Further studies have indicated that andrographolide beneficially modulates the gut microbiota, potentially influencing the levels of neuroactive microbial metabolites such as SCFAs. The cAMP-PKA-CREB-HDAC3 signaling pathway, which is downstream of SCFAs, is activated in microglia from AD mice, and andrographolide can inhibit the activity of this pathway (Zhang S. et al., 2022).

Baicalin, a bioactive flavonoid compound extracted from Scutellaria baicalensis, can improve gut barrier function by regulating microbial diversity, increasing the abundance of probiotics (e.g., lactobacilli), and reducing pro-inflammatory bacteria (e.g., *E. coli*), thereby increasing SCFA production. The resulting SCFAs cross the BBB and activate gut–brain axis signaling (e.g., via the vagus nerve or humoral pathways), further inhibiting Aβ aggregation and tau hyperphosphorylation, thereby exerting a therapeutic effect on AD (Zhang S. et al., 2022; Hu et al., 2023).

Glycyrrhizin (GS) is a natural isoflavone that demonstrates multi-target therapeutic potential in AD by modulating the gut–brain axis. As a phytoestrogen precursor, GS can be metabolized by specific symbiotic bacteria (e.g., *Adlercreutzia equolifaciens*) into (S)-equol. The latter enhances intestinal barrier function by activating estrogen receptor *β* (ERβ) and inhibits the colonization of pathogenic bacteria (e.g., *Escherichia coli*), thereby reducing systemic inflammation (Loew et al., 2024). Notably, the combination of GS and epigallocatechin gallate (EGCG) synergistically blocks pro-inflammatory signaling pathways (e.g., the NLRP3 inflammasome) and reduces neuroinflammation (Muraleedharan and Ray, 2024).

One study (Liu et al., 2025) used D-galactose-induced aged mice as a model to investigate whether Chimonanthus salicifolius flavonoids (CsFE) delay brain aging by modulating the gut microbiota and its metabolites. The results showed that CsFE significantly increased the abundance of beneficial bacteria, including Akkermansia, Muribaculaceae, Lactobacillus, and Lachnospiraceae (e.g., Akkermansia abundance increased approximately 2.5-fold), and promoted the production of short-chain fatty acids (SCFAs; total SCFA levels increased by about 40%). These changes were associated with improved cognitive function, reduced neuroinflammation and oxidative stress, and protection of synaptic function.

### 6.4 Saponins

Panax notoginseng saponins (PNS) are the primary active components of the TCM herb Panax notoginseng. Animal studies have shown that PNS can reverse dysbiosis induced by a high-fat diet or drugs (e.g., scopolamine), reduce the abundance of harmful bacteria such as *Escherichia* and *Clostridium*, and simultaneously increase the abundance of beneficial bacteria such as *Bacteroides* (Park and Wu, 2022). By activating G protein-coupled receptors (GPR41/GPR43) via the microbiota–short-chain fatty acid (e.g., butyrate) pathway, PNS suppresses neuroinflammation and enhances BBB integrity. Vagus nerve section experiments confirm that part of the neuroprotective effect of PNS depends on signaling via the gut–brain axis (Park and Wu, 2022). Other studies have shown that PNS reduces the entry of intestinal LPS into the bloodstream, blocks the TLR4/NF-κB signaling pathway, and lowers levels of IL-1β and TNF-*α* in the brain (Pavlov et al., 2025).

The benefits of ginsenosides in treating AD stem largely from their systemic regulation of the gut–brain axis. Studies have found that ginsenosides (e.g., ginsenoside Rd) and fermented red ginseng can effectively suppress the expression of pro-inflammatory factors such as interleukin-6 (IL-6) and TNF-α in the colon, thereby interrupting the chain of inflammation transmission from the gut to the brain (Shin et al., 2023). At the same time, ginsenosides can reduce the production of harmful metabolites by correcting dysbiosis and promote the production of neuroprotective substances (e.g., SCFAs) by beneficial bacteria (Shin et al., 2023). Further research has shown that ginsenosides can reduce blood levels of cortisol (a stress hormone) and indirectly stabilize HPA axis function by regulating the gut microbiota, thereby alleviating stress-related neurological damage (Shin et al., 2023).

Studies have shown that ICA can significantly reshape the gut microbiome, enriching beneficial bacteria (such as SCFA-producing genera) and reducing harmful bacteria (such as ammonia-producing bacteria) (Li S. et al., 2025; Yi et al., 2026). By increasing levels of SCFAs (e.g., butyrate and propionate), ICA improves intestinal barrier function and reduces the spread of systemic inflammation to the central nervous system (Luo et al., 2025) (Table 1).

**Table 1:** Summary of mechanisms of active ingredients from TCM treating AD via the gut-brain axis.

Active ingredients in TCM	Source	Target microbiota/metabolites	Molecular mechanism	Neuroprotective effects	References
Eucommia bark polysaccharides	*Eucommia ulmoides* Oliv. (Eucommiaceae)	Increases SCFA-producing bacteria (*Butyricicoccus*, Lactobacillaceae, Firmicutes) and butyrate; reduces *Helicobacter* and *Clostridium*	Activates GPR41/43 to inhibit NF-κB; upregulates tight junction proteins (occludin, claudin-1, ZO-1); promotes M2 microglial polarization; regulates glutamine synthetase to enhance autophagy	Reduces neuroinflammation; improves cognition; promotes Aβ clearance; upregulates TGF-β and BMP-6	Peng et al. (2024), Zhang et al. (2025), and Li Y. et al. (2024)
Pseudostellaria heterophylla polysaccharide	Pseudostellaria heterophylla (Miq.) Pax (Caryophyllaceae)	Increases *Lactobacillus*, *Muribaculum*, *Monoglobus*, and *Lactococcus siraeum* group; reduces UCG-009 and *Blautia*	Repairs intestinal barrier; lowers peripheral inflammatory cytokines	Promotes M1 → M2 and A1 → A2 conversion; improves cognition	He C. et al. (2024)
Asparagus polysaccharides	Asparagus cochinchinensis (Lour.) Merr. (Asparagaceae)	Increases *Lactobacillus gasseri* and Lachnospiraceae; reduces pathogenic bacteria	Restores microbial metabolic functions (bile acid and SCFA synthesis)	Prevents and alleviates AD pathology	Chen et al. (2023)
Berberine	Coptis chinensis Franch. (Ranunculaceae)	Increases SCFA-producing bacteria (*Butyricimonas*, *Ruminococcus*, *Bifidobacterium*) and acetate/indole-3-lactic acid; reduces LPS-producing *Arcobacter*	Inhibits HDAC; activates GPR41/43 to suppress NF-κB; upregulates mucin-2 and tight junctions	Attenuates microglial activation and Aβ-induced neurotoxicity; lowers IL-1β and TNF-*α*; improves spatial memory	Xie et al. (2023) and Sun C. et al. (2024)
Matrine	*Sophora flavescens* Aiton (Fabaceae)	Restores microbial diversity; increases beneficial bacteria; reduces pathogens, serum DAO and LPS	Inhibits kynurenine pathway (reducing quinolinic acid) and TLR4/NF-κB signaling	Protects neurons; lowers neurotoxic metabolites and intestinal permeability; reduces TNF-*α* and IL-1β	Zhang et al. (2023), Deng et al. (2021), Bao et al. (2022), and Tiwari and Paramanik (2025)
Palmatine	Coptis chinensis Franch. (Ranunculaceae)	Increases SCFA-producing bacteria (*Ruminococcus*, *Bifidobacterium breve*) and acetate/indole-3-lactic acid	Activates AMPK/NF-κB pathway via BBB or vagus nerve	Suppresses neuroinflammation; reduces Aβ deposition	Xie et al. (2023)
Andrographolide	*Andrographis paniculata* (Burm.f.) Wall. ex Nees (Acanthaceae)	Modulates gut microbiota and neuroactive metabolites (e.g., SCFAs)	Inhibits cAMP-PKA-CREB-HDAC3 signaling downstream of SCFAs	Reverses cognitive deficits; reduces Aβ plaques and neuroinflammation in APP/PS1 mice	Zhang S. et al. (2022)
Baicalin	Scutellaria baicalensis Georgi (Lamiaceae)	Increases *Lactobacillus* and SCFAs; reduces *E. coli*	Promotes GLP-1 secretion via TGR5; inhibits microglial overactivation; upregulates SOD	Suppresses Aβ aggregation and tau hyperphosphorylation; lowers IL-6 and TNF-α; attenuates oxidative stress	Zhang S. et al. (2022) and Hu et al. (2023)
Glycyrrhizin	*Glycyrrhiza glabra* L. (Fabaceae)	Metabolized by *Adlercreutzia equolifaciens* to (S)-equol	Activates ERβ to strengthen intestinal barrier; inhibits *E. coli* colonization; synergizes with EGCG to block NLRP3 inflammasome	Reduces systemic and neuroinflammation	Loew et al. (2024) and Muraleedharan and Ray (2024)
Panax notoginseng saponins	Panax notoginseng (Burkill) F. H. Chen (Araliaceae)	Increases *Bacteroides* and butyrate; reduces *Escherichia* and *Clostridium*	Activates GPR41/43 to inhibit NF-κB; blocks TLR4/NF-κB; lowers brain IL-1β and TNF-α	Suppresses neuroinflammation; enhances BBB integrity	Pavlov et al. (2025), Park and Wu (2022), and Pavlov et al. (2025)
Ginsenosides	*Panax ginseng* C. A. Mey. (Araliaceae)	Corrects dysbiosis; promotes SCFA production by beneficial bacteria	Suppresses colonic IL-6 and TNF-α; reduces serum cortisol; stabilizes HPA axis	Interrupts gut-brain inflammatory cascade; alleviates stress-induced neuronal damage	Shin et al. (2023)
Icariin	Epimedium sagittatum (Siebold & Zucc.) Maxim. (Berberidaceae)	Enriches SCFA-producing genera; reduces ammonia-producing bacteria; elevates butyrate and propionate	Enhances intestinal barrier via SCFAs	Limits systemic inflammation spreading to CNS	Li S. et al. (2025), Yi et al. (2026), and Luo et al. (2025)
Curcumin	*Curcuma longa* L. (Zingiberaceae)	Increases *Lactobacillus*	Modulates NF-κB pathway	Reduces neuroinflammation; modulates gut microbiota	Pavlov et al. (2025) and Kim et al. (2024)
Ginkgolides	*Ginkgo biloba* L. (Ginkgoaceae)	Modulates immune responses in gut-brain axis	Activates BDNF/CREB pathway	Enhances synaptic plasticity	Pavlov et al. (2025)
Ganoderma triterpenes	Ganoderma	Regulates gut microbiota and aromatic amino acid metabolism	Inhibits oxidative stress	Attenuates oxidative damage	Shen et al. (2024)
Resveratrol	*Reynoutria japonica* Houtt. (Polygonaceae)	-	Regulates Th1/Th2 balance	Reduces systemic inflammation	Kim et al. (2024), Gu et al. (2024), and Fang et al. (2026)

### 6.5 Other

The active constituents of TCM are not limited to the categories mentioned above. Phenolic acids can act as “prebiotics,” selectively promoting the growth of beneficial gut bacteria (e.g., *Bifidobacterium* and *Lactobacillus*) while inhibiting the proliferation of potentially pathogenic bacteria, thereby correcting the gut microbiota dysbiosis associated with AD (Wang Z. et al., 2026; Shabbir et al., 2021; Zhang Y. et al., 2022). Many phenolic compounds (e.g., lignans) are not easily absorbed directly but can be metabolized by specific gut bacteria into compounds with higher biological activity and greater bioavailability (e.g., lactones and lactols). These microbial metabolites not only enhance their own neuroprotective activity but also penetrate the BBB more effectively (Wang Z. et al., 2026; Reddy et al., 2020).

**Figure 1:**
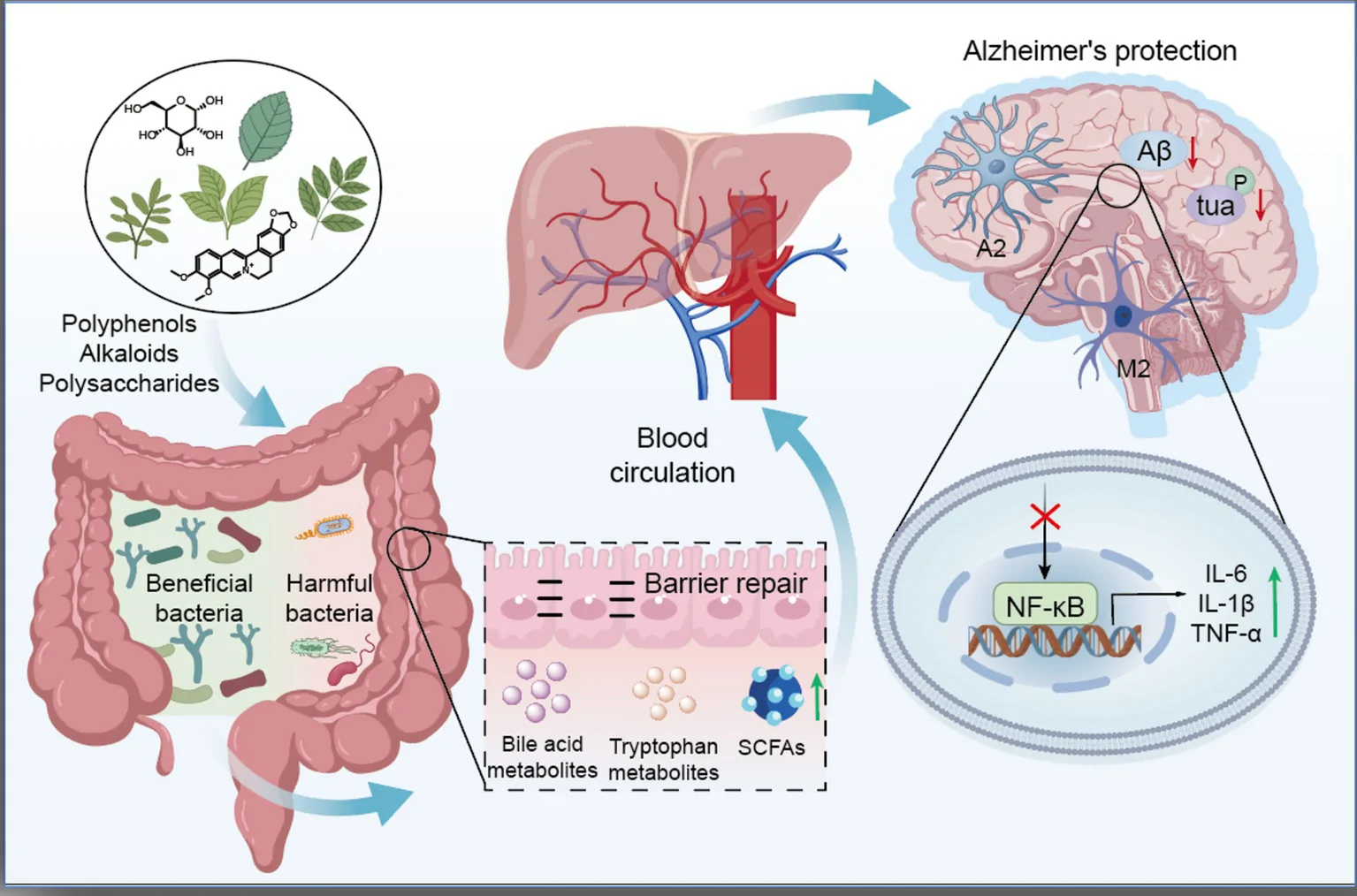
Gut-microbiota-brain axis: natural products targeting microbiota metabolism and signaling to improve Alzheimer’s disease.

By regulating the gut microbiota, phenolic acids also promote the production of beneficial metabolites such as SCFAs in the gut. These SCFAs help maintain the integrity of the intestinal epithelial barrier, prevent LPS from entering the bloodstream and triggering systemic inflammation, stabilize the BBB, and reduce the impact of peripheral inflammatory factors on the brain (Zhang Y. et al., 2022; Ticinesi et al., 2022; Mustafa et al., 2025). Take EGCG as an example: this major bioactive polyphenol found in green tea promotes the growth of beneficial bacteria such as *Bifidobacterium* and *Lactobacillus*, inhibits pathogenic bacteria, improves intestinal barrier function, restores microbial diversity, reduces the release of pro-inflammatory factors, and indirectly suppresses neuroinflammation (Muraleedharan and Ray, 2024; Wijeweera et al., 2023). Notably, EGCG can be converted by gut microbiota into metabolites with higher biological activity (e.g., SCFAs), which influence brain function via the bloodstream or vagal nerve signals (Wang Z. et al., 2026) (Figure 1).

### 6.6 Common methodological limitations across current studies

Despite the coherent mechanistic framework described above, the following limitations are prevalent and impede causal inference and clinical translation.

Correlation without causation. Most studies report associations between TCM-induced microbiota changes and cognitive outcomes. Gold-standard causal tests—such as fecal microbiota transplantation from TCM-treated donors to germ-free AD mice, GPR41/43 knockout, or selective depletion of SCFA-producing bacteria—are rarely performed.

Absolute SCFA concentrations are rarely reported. The majority of studies present SCFA changes as fold-changes or relative abundances, without absolute values (e.g., μmol/g cecal content or μM in plasma). This makes it impossible to compare with *in vitro* effective concentrations (e.g., 100–300 μM for acetate) or to assess physiological relevance.

Overreliance on LPS-stimulated microglia *in vitro*. Although AD neuroinflammation is chronically driven by sterile stimuli (Aβ, tau, myelin debris), most in vitro mechanistic validation uses acute LPS stimulation. As detailed in Section 7, this may not recapitulate the relevant pathology.

Neglect of inter-individual variability. Compounds such as berberine require gut microbial conversion to active metabolites. Human microbiota composition varies greatly among individuals, yet this is rarely modeled in preclinical studies.

Single-compound focus versus TCM formulas. The majority of studies isolate single ingredients, while TCM is often used as multi-herb formulas. Potential synergistic effects on the gut–brain axis are therefore overlooked.

Species differences between animal models and humans. The vast majority of the above studies use transgenic or chemically induced mouse models of AD, which cannot fully recapitulate the complex pathophysiology of human AD (e.g., long disease course, multiple comorbidities, genetic heterogeneity). Moreover, significant differences exist between mice and humans in gut microbiota composition, immune system, and drug-metabolizing enzymes. Therefore, the efficacy and mechanisms observed in animal models may not be directly replicable in humans. Future clinical studies (especially randomized controlled trials) and validation using humanized microbiota mouse models are needed to bridge this translational gap.

These limitations are not insurmountable; however, future research must address them to move the field from descriptive correlation toward mechanistic validation and ultimately clinical translation.

## 7 Integrated analysis of mechanisms of action

Based on the aforementioned studies of various bioactive components in TCM, their mechanisms of action in treating AD via the gut–brain axis can be integrated into three levels: gut microbiota restructuring, regulation of gut microbial metabolism, and modulation of gut–brain axis signaling. These three mechanisms do not operate in isolation but are instead intertwined and synergistic, forming a multi-level intervention network that spans from the gut microbiome to central nervous system function.

### 7.1 Cascade coupling of mechanistic pathways

Microbiota restructuring is a prerequisite for metabolic regulation.

The composition of the gut microbiota determines the types and abundance of metabolic products. An increase in the abundance of SCFA-producing bacteria (e.g., Butyricicoccus, Lactobacillus, and Bifidobacterium) directly boosts the production of SCFAs such as butyrate and propionate. Concurrently, a reduction in pathogenic bacteria (e.g., LPS-producing Arcobacter species) lowers the endotoxin burden and alleviates metabolic stress. This structural shift—characterized by an increase in beneficial bacteria and a decrease in pathogenic bacteria—lays the foundation for subsequent metabolic effects. Gut barrier repair (via upregulation of tight junction proteins) serves as the physical safeguard for this remodeling, ensuring the retention of beneficial metabolites and the exclusion of harmful ones.

Metabolites serve as the central hub linking the microbiota to host signaling. As key effector molecules, SCFAs play a triple role as energy substrates, signaling molecules, and epigenetic modulators: they provide energy to intestinal epithelial cells to maintain barrier integrity; they cross the BBB to directly nourish central nervous system neurons; and they activate GPR41/43 receptors to inhibit the NF-κB pathway, thereby bridging metabolic regulation and immune suppression. Tryptophan metabolites (serotonin vs. quinolinic acid) and bile acid metabolites similarly act as bridges, converting microbial enzymatic reactions into chemical signals recognizable by the host.

Signal regulation integrates metabolic inputs and outputs to mediate neuroprotection. Metabolic signaling interacts with neuroendocrine and immune-inflammatory pathways in a bidirectional manner: SCFAs activate GPR receptors to inhibit NF-κB, regulating inflammation in parallel with HDAC inhibition; stable HPA axis function mitigates stress-induced damage to the microbiota, creating a protective feedback loop; and the BDNF/CREB pathway integrates nutritional signals and synaptic activity, serving as the final integration point for the metabolic–neural dialogue.

### 7.2 Cross-talk and synergistic amplification among pathways

#### 7.2.1 The central role of SCFAs

SCFAs serve as core nodes in the network, intersecting with multiple pathways:

Butyrate interacts with multiple physiological processes: it promotes intestinal epithelial health to influence tryptophan absorption and local metabolism; it jointly regulates energy metabolism and insulin sensitivity in conjunction with bile acid metabolism; it suppresses both peripheral and central inflammation via the GPR41/43 receptor system; and it provides energy to neurons while regulating microglial phenotype, thereby supporting neurogenesis.

#### 7.2.2 The inflammation-metabolism-neuroscience triad

Changes in metabolites, inflammatory states, and neural function form a closed loop. LPS entering the bloodstream activates the TLR4/NF-κB pathway (Sun Y. et al., 2024), thereby exacerbating neuroinflammation, which in turn, via the vagus nerve, downregulates intestinal motility and secretion. Conversely, SCFAs inhibit NF-κB, breaking this vicious cycle while promoting the production of anti-inflammatory factors such as IL-10, thus establishing a protective positive feedback loop.

#### 7.2.3 Neuroendocrine modulation

As the central hub of the body’s stress response, the HPA axis modulates the entire network. Chronic stress elevates corticosterone levels, which damages the hippocampus while simultaneously altering intestinal blood flow and barrier function. Meanwhile, metabolite signals (e.g., SCFAs) influence the hypothalamus via the vagus nerve, regulating HPA axis activity and facilitating bidirectional gut–brain communication.

### 7.3 Integrative characteristics of a multi-level intervention network

The core characteristic of this network is bidirectional adaptability: peripheral metabolic states are reflected in real-time in central functions, while central demands regulate the peripheral environment through neurohumoral pathways. Through multi-point intervention (i.e., targeting microbiota, metabolism, and signaling), TCM components break the vicious cycle associated with pathological states and restore a protective dynamic equilibrium.

### 7.4 The pathophysiological significance of mechanism integration

Dysregulation of the gut–brain axis in AD manifests as multi-level disruptions along the microbiota–metabolism–signaling axis: a reduction in SCFA-producing bacteria, decreased levels of protective metabolites such as butyrate, disruption of the intestinal barrier, entry of LPS into the bloodstream, systemic inflammation leading to microglial activation, impaired Aβ clearance, tau hyperphosphorylation, and cognitive decline. The value of TCM intervention lies in its ability to simultaneously correct abnormalities across multiple pathways rather than targeting isolated points. For example, berberine simultaneously increases butyrate-producing bacteria, reduces LPS-producing bacteria, and upregulates tight junction proteins, exerting effects at all three levels. Similarly, EUPs promote SCFA production, repair the intestinal barrier, and suppress neuroinflammation, creating a cascading amplification effect.

### 7.5 Synergistic effects of TCM herbal formulas

The clinical practice of TCM herbal formulas suggests that multi-herb formulations may achieve therapeutic effects against diseases through more complex synergistic mechanisms (You et al., 2020; Huang et al., 2024; Liu et al., 2023; Xu et al., 2022; Zhao et al., 2021; Zhu et al., 2023). Taking Bushen Yinao Pill as an example, randomized controlled trials have confirmed that it improves cognitive function in AD patients by modulating gut microbiota composition (increasing beneficial bacteria, reducing harmful bacteria) and lowering peripheral inflammatory burden (downregulating Aβ, IL-6, and TNF-*α*). Unlike single compounds that typically target a limited set of microbial taxa or signaling pathways, the diverse active ingredients in formulas—alkaloids, flavonoids, saponins, polysaccharides, and others—can simultaneously act on multiple nodes: microbiota restructuring (increasing SCFA-producing bacteria, reducing pathogens), metabolic regulation (elevating protective metabolites such as SCFAs and bile acids), barrier repair (upregulating tight junction proteins), and neuroimmune signaling (inhibiting NF-κB, activating BDNF/CREB). This “multi-point initiation, network-level modulation” paradigm may be more effective than single compounds in breaking the vicious cycle of AD pathology and restoring systemic homeostasis of the gut–brain axis. Based on the above preliminary evidence, future studies should incorporate longitudinal dynamic monitoring and causal validation strategies (e.g., microbiota transplantation or selective microbial modulation) to establish the temporal hierarchy and causal links among microbiota remodeling, metabolic regulation, barrier repair, and neuroimmune signaling. Such an approach would systematically decode how TCM formula-derived active ingredients collectively orchestrate gut–brain homeostasis.

**Figure 2:**
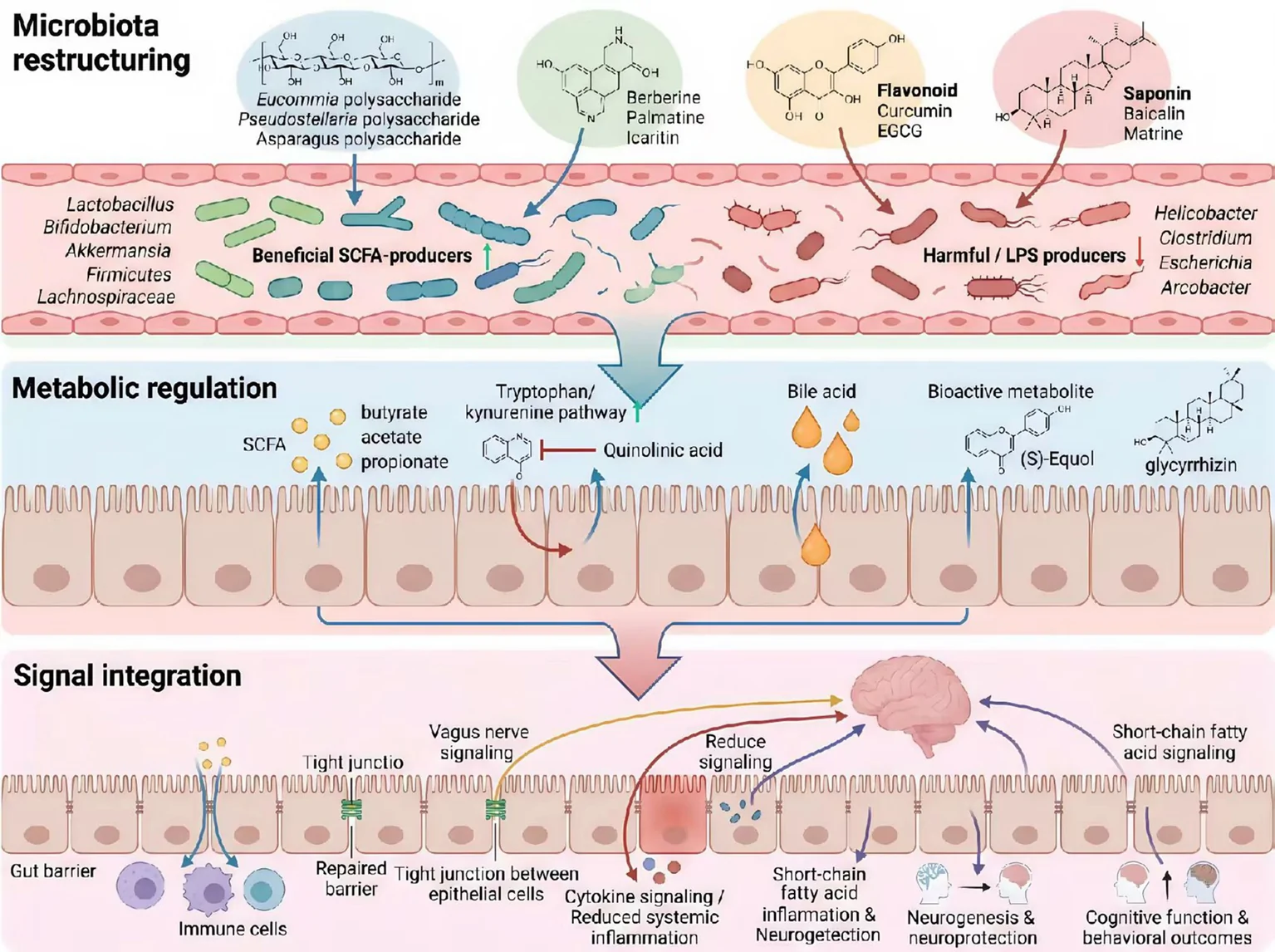
Schematic diagram of the multi-target intervention mechanism on the Gut-brain axis based on the “structure-metabolism-signaling” cascade.

In summary, the active components of TCM establish a dynamic, multi-target, adaptive intervention network through “structure–metabolism–signal” cascade coupling and “gut–periphery–brain” cross-level integration, thereby achieving systemic regulation of AD pathology. The analysis in this section is synthesized from the following references and those cited in the preceding text (Zhao J. et al., 2026; Pavlov et al., 2025; Long et al., 2024; Zhou et al., 2025; Liang et al., 2023; Ma et al., 2023; Wang H. et al., 2025; Mo et al., 2025; Yin et al., 2025; Xue et al., 2026; Fu et al., 2023; Fu et al., 2026; Wang et al., 2024; Mana et al., 2025; Wang T. et al., 2025; Wu et al., 2025; Deng et al., 2022) (Figure 2).

## 8 Mechanism paradoxes and validation dilemmas

The preceding sections systematically reviewed the integrated mechanism by which active components of TCM produce SCFAs via the gut microbiota, thereby inhibiting neuroinflammation and improving AD pathology. Most studies in this field assume that SCFAs (acetate, propionate, and butyrate) possess anti-inflammatory effects, and *in vitro* experiments commonly use LPS-stimulated microglia as a model of neuroinflammation. However, notable contradictions exist between the results of different studies.

It has been reported in the literature (Yang et al., 2026) that the anti-inflammatory effects of acetate in EOC20 mouse microglia are stimulus-dependent: under LPS stimulation, acetate at physiological concentrations (100–300 μM) failed to reduce pro-inflammatory cytokine secretion or inhibit inducible nitric oxide synthase (iNOS) expression; however, in a mechanical stretching model (simulating traumatic brain injury), acetate at the same concentrations significantly inhibited TNF-*α* production and NF-κB nuclear translocation. Furthermore, supraphysiological concentrations of acetate exhibited no anti-inflammatory effects. In other words, acetate is effective against biomechanical stimulation but ineffective against LPS stimulation.

Another study in aged mice (23–24 months old) found (Vailati-Riboni et al., 2022) that, compared with adult mice, aged mice had significantly lower levels of cecal SCFAs (acetate, butyrate, and propionate), while microglia exhibited an activated phenotype and increased TNF-*α* secretion. Following administration of an inulin-rich diet, cecal SCFA levels recovered, and the gene expression profile of microglia and TNF-α secretion returned to levels observed in adult mice. These results suggest that increasing endogenous SCFAs through dietary fiber can reverse age-related microglial dysfunction.

The findings of these two studies present an apparent paradox: while acetate is ineffective *in vitro* under LPS stimulation, elevating SCFAs (including acetate) in aged mice exhibits anti-inflammatory effects. Possible explanations for this discrepancy include the following. First, differences between in vitro and *in vivo* models. *In vitro* LPS stimulation represents acute, high-intensity TLR4 activation, which may mask the mild modulatory effects of SCFAs; chronic, low-grade inflammation in aged mice is driven by multiple factors, including dysbiosis, intestinal barrier damage, and altered metabolites. SCFAs may exert their effects through indirect pathways, such as repairing the intestinal barrier and modulating peripheral immunity, rather than acting directly on microglia. Second, specificity of SCFA types. Inulin elevates a mixture of multiple SCFAs, whereas butyrate has been extensively shown to directly inhibit LPS-induced microglial activation; the conclusions of the aforementioned *in vitro* studies are limited to acetate and cannot be extrapolated to other SCFAs. Third, matching of stimulus types. In the aging brain, microglial activation stems more from endogenous damage-associated molecular patterns (Aβ, tau, myelin fragments) than from LPS; acetate is effective against aseptic injury (mechanical stretching), suggesting it may have anti-inflammatory potential in pathologies such as AD, which are primarily characterized by aseptic inflammation. Fourth, unclear dosage. The effective concentration of acetate *in vitro* is 100–300 μM, which is close to physiological levels; however, the absolute concentration of SCFAs in the cecum following inulin intervention has not been reported, making it impossible to determine whether it falls within the effective range.

The aforementioned paradox highlights several validation challenges in current gut–brain axis research. First, the anti-inflammatory effects of SCFAs should not be treated as a universal conclusion; distinctions must be made based on specific SCFA types, types of inflammatory stimuli, and dosage ranges. Second, the correlation observed *in vivo* between elevated SCFA levels and reduced neuroinflammation requires causal validation through specific blockade experiments (e.g., GPR41/43 knockout, vagotomy, or depletion of SCFA-producing microbiota). Third, future studies should report the absolute concentrations of individual SCFAs in the gut or plasma following TCM or dietary fiber interventions, and compare these with effective concentrations *in vitro*. Fourth, microglial models using AD-relevant stimuli (Aβ, tau) should be prioritized to validate the anti-inflammatory activity of target SCFAs, rather than assuming that results from LPS models are universally applicable.

To resolve this paradox and directly test the hypothesis that acetate’s anti-inflammatory effects are diminished under Aβ-rich conditions, we propose two complementary validation strategies:

Validation Strategy 1: Multi-cell co-culture system — Establish co-culture systems of microglia with astrocytes and neurons to simulate the AD brain microenvironment. Under Aβ stimulation, treat the co-cultures with physiologically relevant concentrations of acetate and evaluate its inhibitory effects on the NF-κB pathway and NLRP3 inflammasome, and compare with the LPS stimulation group. This strategy can determine whether Aβ renders microglia resistant to acetate’s anti-inflammatory effects, and whether other brain cell types alter this response through paracrine signaling.

Validation Strategy 2: Dose–response study in AD transgenic mouse models — Conduct acetate intervention experiments in AD model mice, systematically detecting dose–response relationships between brain SCFA concentrations, microglial activation status, neuroinflammatory markers, and cognitive function. This strategy can determine whether increasing systemic SCFA levels can overcome the Aβ-associated resistance observed *in vitro*, and identify the threshold concentrations required for therapeutic benefit *in vivo*.

The combination of these two strategies will determine whether the SCFA paradox reflects a genuine limitation of acetate-based interventions in AD, or whether compensatory mechanisms in vivo can bypass the impaired direct anti-inflammatory signaling observed in isolated microglial cultures.

In summary, the existing literature is not truly contradictory; rather, it collectively indicates that the effects of SCFAs are stimulus-dependent, species-specific, and dose-dependent. The simplified assumption widely adopted in the field—that “SCFAs are universally anti-inflammatory and that the LPS model represents all cases”—is precisely the root cause of the aforementioned paradox. The overall benefits of dietary fiber intervention *in vivo* may stem from the synergistic effects of multiple SCFAs and indirect mechanisms such as intestinal barrier repair, rather than the direct action of a single SCFA on microglia. This paradox does not negate the gut–brain axis hypothesis; rather, it suggests that the field needs to move from descriptive correlations toward mechanistic validation, breaking away from overreliance on LPS models and the assumption of universal anti-inflammatory effects of SCFAs.

## 9 Conclusion

In summary, gut microbiota dysbiosis and subsequent gut–brain axis dysregulation are key pathological drivers of AD. Bioactive components of TCM—polysaccharides, alkaloids, flavonoids, saponins, and others—can intervene in this process through three integrated and synergistic levels: restructuring gut microbial communities (increasing SCFA-producing bacteria and reducing pathogenic taxa), regulating microbial metabolites (especially SCFAs, tryptophan derivatives, and bile acids), and modulating gut–brain signaling pathways (including GPR41/43, NF-κB, HPA axis, and BDNF/CREB). This multi-level network, characterized by “structure–metabolism–signal” cascade coupling and “gut–periphery–brain” cross-level integration, enables TCM constituents to break the vicious cycle of AD pathology and restore a protective dynamic equilibrium.

Importantly, this review goes beyond a descriptive summary of beneficial effects. We explicitly address a central paradox in the field: the anti-inflammatory action of SCFAs is not universal but rather stimulus-dependent, type-specific, and dose-dependent. The common assumption that “SCFAs are universally anti-inflammatory and LPS-stimulated microglia represent all neuroinflammatory contexts” is an oversimplification that underlies many current contradictions. As discussed in Section 8, acetate’s anti-inflammatory effects may be compromised in Aβ-rich environments, necessitating experimental validation through multi-cell co-culture systems and dose–response studies in AD mouse models.

To address these challenges, we outline the following research roadmap that systematically links current methodological gaps to specific remedial approaches:

- Correlation without causation: Longitudinal cohort studies combined with Mendelian randomization or gnotobiotic transplantation models should be employed to establish causal relationships between gut microbiota changes and AD pathology.
- Unreported absolute SCFA concentrations: Metabolomics protocols should be standardized to report absolute SCFA levels (e.g., μmol/g cecal content, μM plasma) and compare with *in vitro* effective concentrations (100–300 μM for acetate).
- Overreliance on LPS models: AD-relevant stimuli (Aβ, tau, myelin debris) should be prioritized in microglial assays, and chronic low-intensity stimulation protocols that better mimic AD pathophysiology should be developed.
- Neglect of inter-individual variability: Microbiome diversity profiling should be incorporated in preclinical studies, and stratified clinical trials based on gut microbiota composition should be conducted.
- Single-compound focus: Network pharmacology should be applied to investigate synergistic effects in TCM formulas, and head-to-head comparisons between isolated compounds and multi-herb formulations should be performed.
- Species differences: Humanized microbiota mouse models should be utilized, and randomized controlled trials should be initiated to validate clinical translation across species.
- Water solubility and bioavailability: Novel strategies including nanocarriers, structural derivatization and co-delivery platforms ought to be exploited to elevate the aqueous solubility and targeting property of TCM bioactive ingredients (e.g., berberine, curcumin), whose poor water solubility leads to low oral bioavailability and restricted clinical efficacy (Duan et al., 2024; Chen et al., 2021; Zhang et al., 2024; Yu et al., 2022).
- By addressing these challenges through rigorous causal testing, standardized reporting, and translational validation, TCM-based gut–brain axis strategies hold significant promise for developing effective AD therapies.

## Glossary

Aβ: Amyloid beta
AD: Alzheimer's disease
BBB: blood-brain barrier
BBR: Berberine
CUMS: chronic stress
DAO: diamine oxidase
EGCG: epigallocatechin gallate
ERβ: estrogen receptor β
EUPs: Eucommia bark polysaccharides
FDA: U.S. Food and Drug Administration
GLP-1: glucagon-like peptide-1
GPR41/GPR43: G protein-coupled receptors
GS: Glycyrrhizin
HDACs: histone deacetylases
ICA: Icariin
ILA: indole-3-lactic acid
iNOS: inducible nitric oxide synthase
Kyn: kynurenine
LPS: lipopolysaccharide
PH-PS: Pseudostellaria heterophylla polysaccharide
PNS: Panax notoginseng saponins
ROS: reactive oxygen species
SCFAs: short-chain fatty acids
tau: Tau protein
TCM: traditional Chinese medicine
WHO: World Health Organization
